# A Comparative Study to Assess the Accommodation and Vergence Relationship of Myopia in Indian Adolescent

**DOI:** 10.4314/ejhs.v33i3.16

**Published:** 2023-05

**Authors:** Sadiya Ikram Syeda, Radha Kumar, Xavier C Jayaseelan, Rajagopalan Vijayaraghavan

**Affiliations:** 1 Lecturer-Optometry, Department of Ophthalmology, Saveetha Medical College & Hospitals, Chennai, Tamil Nadu, India; 2 Professor, Department of Paediatrics, Saveetha Medical College & Hospitals, Chennai, Tamil Nadu, India; 3 Professor, Department of Ophthalmology, Saveetha Medical College & Hospitals, Chennai, Tamil Nadu, India; 4 Director Research, Department of Research, Saveetha Medical College & Hospitals, Chennai, Tamil Nadu, India

**Keywords:** Myopia, Vergence, Accommodation, Binocular vision

## Abstract

**Background:**

Accommodation and Vergence disorder are diverse visual anomalies which can interfere with a child's school performance and impair one's ability to function efficiently. Its association with refractive error and its intervention were studied less in Indian myopia children; hence, there is a need for research in such setting.

**Method:**

One hundred and fifty Indian adolescents aged 10 to 17 years were divided into three refractive error groups (high, moderate, and low myopia). Baseline vision examination and a comprehensive binocular vision assessment were performed on all eligible adolescents. Vision therapy was provided to participants whose parents gave consent on behalf of the children. Chi-square analysis was utilized to look at the association between the groups of refractive errors. To compare the mean constants of the experimental and control groups, a two-way RM ANOVA was performed.

**Results:**

The most common dysfunction found in low myopia (75.3%), and moderate myopia (54%) was convergence insufficiency. High myopes (62.8%) were found to have combined convergence and accommodative insufficiency followed by accommodative dysfunction (14%) and basic exophoria (6%). In moderate myopia, a significant relationship was found between this dysfunction and refractive error. The experimental group in the overall sample showed statistically significant improvement after vision therapy (P<0.001), in comparison to the control group.

**Conclusion:**

Refractive error is linked to accommodative and convergence insufficiency. Thus, vergence and accommodative impairment must be tested for all myopic children, and vision therapy should be advised along with spectacle prescription for efficient binocular vision.

## Introduction

Binocular vision (BV) evaluation is a crucial component of the research of visual systems as effective reading abilities in both children and adults depend on adequate binocular vision (1). The eyes are responsible for organizing and processing more than 80% of perceptual data. Thus, any abnormality in the visual function system has an impact on a child's cognitive growth and academic success (2, 3).

Children's educational attainment has been connected to BV abnormalities. School children do more close work than any other demographic, and the existence of accommodative and binocular impairment can create visual signs that disrupt work and recreational activities. These factors make this population of particular concern (4). Reading proficiency is necessary for academic achievement. Accommodation and vergence processes in the eye need to be precise to read comfortably and easily (5). Most importantly, they may not complain, as they do not realize the need to read comfortably (6).

One of the most common visual disorders in people is myopia. Myopia incidence is alarmingly rising on a worldwide scale (7,8). Understanding how accommodation and convergence function is important since the near visual activity is the foundation of myopia. Over the past decade, many authors have suggested that restoration of clear vision is a critical step in the treatment of non-strabismic persons with accommodative and vergence abnormalities who have a significant refractive error (9–11).

Specific data for BV anomalies among myopic children in Indian literature is not documented. Thus, it becomes appropriate to have data among myopic children with binocular vision anomalies in the clinical decision-making process. It has been stated that NSBVAs (non-strabismic binocular vision abnormalities) are quite common among children nowadays due to the prevalence of mobile phones, computers, and other video consoles (12, 13). Estimates of BV abnormalities in school-aged children will aid in the formulation of an effective intervention, allowing for efficient BV and reading as well as enhancing the children's quality of life in terms of vision.

## Methods

**Figure 1:**
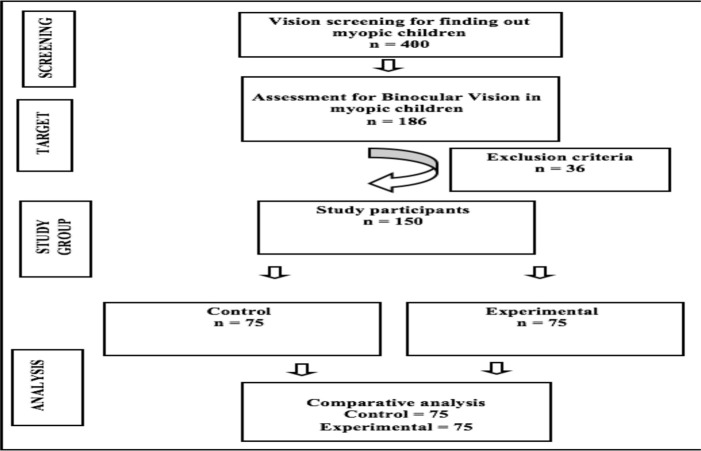
Flow diagram of the study.

**The study participant**: The administration of the school was informed of the specifics of the intended and experimental design, and both the parents' oral and written informed consents were acquired. A total of 400 children aged 10-17 years who were attending the 7-12^th^ grades in a private school in the Chengalpattu district (Tamil Nadu, India) participated in this study. Out of These, 186 students were found to have a myopic refractive error. After determining the sample frame and sampling unit, convenience sampling, using non-probability, was used to enroll 150 students. The flowchart for the requirement of children is depicted in Figure 1.

**Eye examination and vision screening**: Box 1 provides a summary of the steps included in vision screening.

**Box 1:** Vision screening steps

Vision Screening was done with a visual acuity chart with a 6/9 cut-off
Pupillary Examination and Torchlight Examination for gross ocular abnormality
Ocular motility test using Broad H test
W4DT was done for both Distance and Near
Stereo acuity for near using TNO Random Dot stereogram test
Static retinoscopy and Subjective acceptance were done for children with refractive error
Using non-cycloplegic refraction's spherical equivalent power as a basis, children with myopic refractive error who are wearing spectacles with the required refractive correction are divided into three refractive error groups (mild≤2D, moderate≤4D, and extreme myopia.6D). These children are chosen for a thorough evaluation of their binocular vision based on exclusion and inclusion criteria, as well as to identify the most frequent abnormalities in binocular vision based on a dioptric power differential. After getting their consent, children are again grouped into the control and experimental group. Vision therapy of 10 sessions was given to children in the experimental group and a reassessment of binocular vision has been performed for evaluating the improvement. This testing was performed after two weeks of wearing the recommended glasses if it was determined that the children's dioptric differences had increased by more than 0.5D. Refractive error tolerance limitations were taken from the CITT protocol (Convergence insufficiency Treatment Trial) (Scheiman et al., 2005)^12^
Children were sent to the base hospital for treatment of their amblyopia, strabismus, and other ocular abnormalities.

**Inclusion criteria**: Children between the ages of 10 and 17 with best-corrected visual acuity of at least 6/9 and N6, old enough to respond to the subjective concept involved (Clear/Blur, Single/Double), were included.

**Exclusion criteria**: Children with Strabismus, Ocular abnormalities, those who may need prism or added plus lens for near, any case of intraocular/previous squint surgery, self-reported case of head trauma/ocular, and history of juvenile diabetes were excluded.

**Binocular vision assessment protocol**: Binocular vision assessment was done in a room with a standardized illumination level (Minimum of 480 lux), room-length of the standard distance of 6 meters. One hundred and fifty children with myopic refractive error who passed the screening protocol were grouped into three groups based on dioptric power. Children underwent a comprehensive binocular vision assessment, which included Phoria measurement for distance and near Thorington chart, MEM (Monocular estimation method), NPA (Near point of accommodation), NRA (Negative relative accommodation) and PRA (Positive relative accommodation), AF (Accommodation facility) with +/-2.00D accommodative flipper, NPC (Near point of Convergence), PFV (“Positive fusional vergence”) and NFV (“Negative fusional vergence”), VF (Vergence Facility) with 12 base out/3 base-in prism and Interpupillary distance (IPD).

As the MEM Retinoscopy is widely practiced and easy to correlate with clinical findings, it served as a means of determining the person's accommodative condition during close-up visual activity. All of the children's right eyes were examined by scanning over a horizontal meridian while the participants read the paper that had been taped to the retinoscope. The lens power used was recorded which indicates the status of accommodation as a lead or lag.

The crucial factor in identifying accommodating abnormalities is the NPA. The accommodative response may be estimated using a variety of methods, including manual methods like the Push-up approach and the Minus lens approach. The push-up approach, where the near target is put nearer to the eyes until a continuous blur is detected, has been believed to be standard due to simplicity. The near target should be equivalent to or slightly superior to the best corrected near visual acuity. The endpoint of a blur was measured with the ruler centered on the forehead. The test was performed binocularly; three measures for each eye were collected, and the average of the two values was noted in centimeters before being changed to its equivalent in dioptric units.

The range of accommodation at a given distance, from stimulation to relaxation, is measured by PRA and NRA. The minus lenses were then added binocularly in 0.25D steps until the children experienced their first sustained blur, which was noted as PRA. The same procedure was repeated with the plus lenses, and the values were recorded as NRA. The subject fixes at the N6 target or the last line of the near vision chart at a 40cm of distance.

Accommodative facility (AF) testing was done to measure the dynamic of accommodation. In this study, +/-2.00 DS lenses at 40cm were advised for children (13) and were alternatively utilized to cause variations in accommodation. The visual system's responses to accommodation stimulation and relaxation are then recorded. The task is to read the words on a word rock card with the letter N10 as rapidly as one can while switching between plus-minus lenses. In the right eye of each child, a monocular accommodative facility was evaluated first, then a binocular accommodative facility. Two words are represented by one cycle of focusing using the plus and minus lenses.

Using a royal air force ruler with a linear accommodative objective of 6/9 lowered by Snellen's letter, the NPC was evaluated. The tests were repeated thrice and the average was recorded as NPC. Testing for heterophoria is a crucial component of standard optometric examinations, and a Bernell MIM (Muscle Imbalance Measure) card was used to assess the extent of the deviation during the Modified Thorington (MT) test. Several authors have endorsed the MT test because of its control of accommodation, simplicity, high repeatability, and reliability (14, 15). At a distance of 3m and 40cm, respectively, the Maddox rod is positioned in the trial frame in the right eye horizontally for horizontal deviations and vertically for deviations. The children claim that the location of the red streak on the vertical and horizontal numbers indicates the proper prism deviation from the MIM card.

A prism cover test was conducted to determine the degree of heterophoria if the red streak was identified outside of the MIM card or in the event of inconsistent replies. The AC/A ratio was calculated by formula (AC/A=IPD+FD X (NP-FP), where IPD is in centimetre, fixation distance (FD) in meter, far and near phoria (NP, FP) in prism diopters. IPD was evaluated with an IPD ruler (16).

The benefit of utilizing a prism bar to measure fusional vergence amplitudes is that it allows for an objective reassessment of the vergence endpoint based on the one eye deviation during testing (14). To prevent the impact of convergence testing on vergence recovery, the NFV for both near and far will be assessed first, followed by the PFV.

As the test stimulus, a vertical row of letters with a 6/9 Snellen equivalent was used. The number of prisms in front of one eye was gradually increased until the child reported diplopia (fusional vergence break), at which point the number of prisms was decreased until the binocular single vision was recovered (fusional recovery). To replicate the real-world testing conditions in the clinical setting, the vergence testing was carried out in an open space without head support or chin rest.

Testing for vergence facility increases the sensitivity of diagnosing binocular vision abnormalities in addition to fusional vergence amplitudes. Vergence flippers of 12 Base out/3 Base in prisms have been discovered to distinguish the symptomatic from the normal BV group during vergence facility testing, which evaluates the fusional vergence system dynamics (17). The children were instructed to maintain a clean, single vertical row of 6/9 letters while the flip prisms were turned from Base out to in. Base out and base in are flipped once, and the number of full cycles/min was counted. To guarantee bifixation, the simultaneous vergence movement of the eyes was recorded while the test was being conducted. The non-strabismic binocular vision abnormalities (NSBVA) diagnostic criteria were approved from multiple sign system classification by (Scheiman & Wick, 2014) (14), (Hussaindeen et al. (2017) (6).

**Ethics**: The Institutional Ethics Committee of Saveetha Medical College and Hospital Institutional Ethics Committee accepted the research methodology and consent form, (005/08/2021/IEC/SMCH), which follows the concepts outlined in the Declaration of Helsinki, as published in the Indian Journal of Medical Research.

**Statistical software**: Statistical significance was defined as 0.05 or less probability. We conducted statistical analysis and graph plotting using SigmaPlot 14.5 (Systat Software Inc., San Jose, USA).

## Results

Out of 400 screened participants, 150 were entitled and took part in this study. The baseline characteristic is demonstrated in Table 2. Low myopia ≤2D, moderate myopia ≤4D and high myopia ≤6 D compromised 65,50,35 participants respectively. The one-way ANOVA represented that there was a considerable age difference (P<0.001), and non-cycloplegic refraction for the right eye (P<0.001).

**Table 2:** Baseline characteristic

	Low-myopia (n=65)	Moderate- Myopia (n=50)	High- Myopia (n=35)	Total (n=150)	Comparison of 3 groups
**Male, N (%)**	43 (66%)	29 (58%)	18 (51%)	90 (60%)	c2 = 2.180
**Female, n (%)**	22 (34%)	21 (42%)	17 (49 %)	60 (40%)	P = 0.336
**Age, Mean ± SD**	14.6 ± 1.74	14.1 ± 2.31	15.8 ± 1.65	14.8 ±	F = 8.181
	b	c	bc	0.87	P < 0.001
**Non-cycloplegic refraction,**	-1.41 ± 0.53	-2.88 0.51	-4.71 ± 0.45	-3.00 ±	F = 490.661
**Spherical Equivalent,**	ab	ac	bc	1.65	P < 0.001
**Right eye, D, mean ± SD**					

For age and eye refractive error, the same alphabetical characters are mutually significant.

The distribution of NSBVA based on dioptric power difference was significant (c2=22.085; P<0.001) and was summarized in Table 2. Considering the overall sample, the most prevalent abnormalities in low myopia ≤ 2D, moderate myopia ≤ 4D were convergence insufficiency, and high myopes ≤ 6D were found to have combined convergence and accommodative insufficiency. Analysis indicated between post-control and experimental group shows a statistical difference for phoria measurement for distance (P<0.001), phoria measurement for near (P<0.001), AF (P<0.001), Vergence facility (P<0.001) by chi-square analysis as shown in Tables 3 and 4.

**Table 3:** Classification of general binocular dysfunction based on dioptric power group in the overall sample by chi-square analysis

Classification	Low-myopia <2D (n=65)	Moderate-Myopia < 4D (n=50)	High-Myopia < 6D (n=35)	Percentage of occurrence
Accommodative dysfunction				
Accommodative Insufficiency	4	4	2	6.6
Accommodative infacility	1	3	0	2.6
Vergence dysfunction				
Convergence Insufficiency	48	24	11	55.3
Basic Exophoria	1	3	0	2.6
Combination of accommodative and vergence dysfunction				
Accommodative and convergence	11	16	22	32.6
insufficiency				
**n = 150; χ2 = 22.085; P < 0.001**				**100**

**Table 4:** Measurement of Binocular parameters between the control and Experimental group in the overall sample by chi-square analysis

Variable	Pre-test Control/Experimental	Post-test Control/Experimental	Statistical Analysis (All four groups)
**Phoria Measurement for Distance**	χ2 = 0	χ2 = 19.104	χ2 = 42.052
	P = 1.0	P < 0.001	P < 0.001
**Phoria Measurement for Near**	χ2 = 0	χ2 = 18.233	χ2 = 50.957
	P = 1.0	P<0.001	P<0.001
**AF**	χ2 = 2.590	χ2 = 51.550	χ2 = 120.282
	P = 0.459	P < 0.001	P < 0.001
**Vergence Facility**	χ2 = 0.0906	χ2 = 11.563	χ2 = 23.902
	P = 0.763	P < 0.001	P < 0.001

We conducted multiple comparisons of the mean using a two-way RM ANOVA (P≤0.05) with a Bonferroni t-test between the experimental and control group in the overall sample, and the results are shown in Table 5. This was done to find the effectiveness of vision therapy in pre-and post-samples correlating to the significance. The results are reported as mean ±SEM and analyzed with two-way repeated measures (RM) ANOVA for one-factor repetition and Bonferroni's t-test for post-hoc multiple comparisons. Factor A was groups (between-group comparison — Control and Experimental), and Factor B, was tests (within group comparison i.e., repetition factor — Pre-test and Post-test).

**Table 5:** Parameters evaluated (Mean and standard deviation) by two-way RM ANOVA (P<0.05) between Control and experimental group in the overall sample

Parameters	Pre-test Mean± SD	Post-test Mean± SD	Pre-test Mean± SD	Post-test Mean± SD	F	p-value	F	p-value
AC/A ratio	6.0 ± 0.1	6.0 ± 0.1	6.1 ± 0.1	6.0 ± 0.1	F = 0.0515	P = 0.821	F = 6.339	P = 0.013
NPC accommodative target break (in cm)	10.0 ± 0.2	10.0 ± 0.2	11.0 ± 0.2	8.0 ± 0.1	F = 10.307	P = 0.002	F =255.287	P<0.001
NPC accommodative target recovery (in cm)	11.1 ± 0.2	11.1 ± 0.2	12.0 ± 0.2	9.0 ± 0.2	F = 8.454	P = 0.004	F =163.847	P<0.001
AA-right eye (in Diopters)	15.0 ± 0.5	15.2 ± 0.5	14.3± 1.0	16.1 ± 0.5	F =0.00589	P = 0.939	F = 5.030	P=0.026
AA-left eye (in Diopters)	15.0 ± 0.5	15.2 ± 0.5	13.5 ± 0.6	16.4 ± 0.6	F = 0.0663	P = 0.797	F = 200.235	P<0.001
AA-Both eyes (in Diopters)	15.9 ± 0.5	16.0 ± 0.5	14.2 ± 0.6	17.0 ± 0.6	F = 0.186	P = 0.667	F = 134.558	P<0.001
NRA (in Diopters)	3.0 ± 0.1	3.1 ± 0.1	3.3 ± 0.1	4.0 ± 0.1	F = 20.959	P<0.001	F =114.097	P<0.001
PRA (in Diopters)	-4.0 ± 0.2	-4.0 ± 0.2	-4.3 ± 0.2	-5.0 ± 0.2	F = 3.967	P = 0.048	F = 6.199	P=0.014
MEM +Value (in Diopters)	0.944 ± 0.066	0.944 ± 0.066	1.171 ± 0.094	0.951 ± 0.078	F = 1.199	P = 0.276	F = 81.951	P<0.001
MEM -Value (in Diopters)	-1.179 ± 0.101	-1.179 ± 0.101	-1.051 ± 0.044	-0.824 ± 0.032	F = 6.923	P = 0.011	F = 44.913	P<0.001
NFV distance break (in Prism Diopters)	9.040 ± 0.2	9.040 ± 0.2	9.067 ± 0.2	9.520 ± 0.2	F = 0.595	P = 0.442	F = 21.690	P<0.001
NFV distance recovery (in Prism Diopters)	6.787 ± 0.2	6.787 ± 0.2	6.573 ± 0.2	7.147 ± 0.2	F = 0.0559	P = 0.813	F = 14.671	P<0.001
NFV near break (in Prism Diopters	19.627 ± 0.5	19.627 ± 0.5	19.347 ± 0.5	21.480 ± 0.5	F = 1.347	P = 0.248	F = 57.581	P<0.001
NFV near recovery (in Prism Diopters	14.893 ± 0.4	14.947 ± 0.4	14.227 ± 0.4	17.467± 0.4	F = 3.597	P = 0.060	F = 79.052	P<0.001
PFV distance break (in Prism Diopters)	15.027 ± 0.4	15.027 ± 0.4	15.813 ± 0.4	12.373 ± 0.3	F = 21.173	P<0.001	F = 103.671	P<0.001
PFV distance recovery (in Prism Diopters)	6.787 ± 0.2	6.787 ± 0.2	6.573 ± 0.2	7.147 ± 0.2	F = 13.134	P<0.001	F = 81.889	P<0.001
PFV near break (in Prism Diopters)	19.627 ± 0.5	19.627 ± 0.5	19.347 ± 0.5	21.480± 0.5	F = 20.071	P<0.001	F = 66.513	P<0.001
PFV near recovery (in Prism Diopters)	14.893 ± 0.4	14.947 ± 0.4	14.227 ± 0.4	17.467 ± 0.4	F = 19.981	P<0.001	F = 113.292	P<0.001
Vergence facility (in cycles/minute)	10.0 ± 0.2	10.1 ± 0.2	10.0 ± 0.2	13.5 ± 0.2	F = 28.280	P<0.001	F = 342.344	P<0.001
Accommodative facility OD (in cycles/minute)	9.4 ± 0.2	9.6 ± 0.2	9.4 ± 0.2	13.0 ± 0.2	F = 33.744	P<0.001	F = 291.514	P<0.001
Accommodative facility OS (in cycles/minute)	9.7 ± 0.2	9.9 ± 0.2	9.6 ± 0.2	13.1 ± 0.2	F = 27.908	P<0.001	F = 235.850	P<0.001
Accommodative facility OU (in cycles per min)	10.2 ± 0.2	10.4 ± 0.2	10.2 ± 0.2	14.0 ± 0.3	F = 27.840	P<0.001	F = 230.413	P<0.001

“AA: Amplitude of accommodation; NPC: Near point of convergence; AC/A: Accommodation convergence to accommodation, PRA: Positive relative accommodation; NRA: Negative relative accommodation; NFV: Negative fusional vergence; MEM: Monocular estimate method; VF: Vergence facility; PFV: Positive fusional vergence; AF: Accommodation facility”.

Although the two-way ANOVA analysis does not reveal a considerable difference between the pre-and post-test (P = 0.013), the AC/A ratio does vary with the age group. Other parameters such as the monocular estimation method (+VE value P<0.001, -VE value P<0.001), NPC (break values P<0.001, recovery values P<0.001), Amplitude of accommodation (P < 0.001), NRA (P < 0.001), PRA (P = 0.014) and AF (P<0.001), Negative fusional vergence (distance break P<0.001, distance recovery P<0.001, near break P<0.001, near recovery P<0.001 ) and PFV (distance break P<0.001, distance recovery P<0.001, near break P<0.001, near recovery P<0.001), Vergence facility (P<0.001) presented a considerable difference between pre- and post-experimental group.

## Discussion

The findings suggest that vergence and accommodation anomalies play a significant role in refractive error. Correction of a low degree of ametropia helps in stabilizing focus in binocular disorders and is important for improving visual acuity (18). One should not undervalue the impact binocular vision status has on academic performance. If left untreated, abnormalities of binocular vision such as heterophoria and vergence and accommodation disorders may cause problems with reading and writing that will only become worse as students go through their academic careers.

Healthy binocular vision has benefits that go beyond effort and performance in the classroom. Accommodative and vergence difficulties may hinder the academic as well as other extracurricular activities like sports and games. Visual perception abnormalities may also cause difficulties later (19, 20). There are studies indicating that, in myopia, the AC/A ratio is increased. The present study also has shown that there is increase in AC/A ratio, as myopia with higher AC/A ratio may have an imbalance in the comparative potency of vergence and accommodative adaptation (21–25).

The present finding indicates that vergence dysfunction, combined vergence and accommodation, and accommodative dysfunction were commonest. Among these, Convergence insufficiencies were commonest among the low degree myopia, moderate myopia, high myopia and combined dysfunction. Convergence and Accommodation insufficiency was common among the high degree of myopia, moderate myopia, low myopia. It is followed by Accommodative dysfunction and basic exophoria in moderate myopia. The statistics shows that children with modest degrees of myopia had a higher likelihood of developing convergence insufficiency. Regarding convergence insufficiency, previous studies on Chinese teenagers also demonstrated the association with refractive error grouping (4). Following low myopia, high myopia, and intermediate myopia in this analysis, emmetropia had the greatest incidence of convergence insufficiency. It was reported to be 2.5 times higher in the hyperopic and emmetropic groups and 8 times higher in the myopic group in the different research on Chinese school children (26). The relationship between abnormal binocular vision and refractive error is supported by the data from these two investigations. Only two persons with intermediate myopia were included in the research by Wajuihian, which had a sample size of 1056 respondents. The results of the research may have been impacted by the limited sample size of myopic subjects, and it would be challenging to establish a link between refractive error and abnormalities in binocular vision (27).

Although convergence insufficiency was the most often seen result in this research, an analysis of accommodative insufficiency was also conducted since excessive myopia has been associated with a significant co-morbidity rate between the two conditions. In the instance of high myopia, the results revealed a considerable difference in accommodative amplitude, and it was also shown that the symptom of accommodative insufficiency increased with the number of indications of convergence insufficiency. The prevalence of accommodative insufficiency in the research (7.3%) is within the range described in the literature (0.2-17.3%), although it is difficult to compare studies because of the variety of diagnostic criteria (2, 6,25, 28–32).

According to many studies, convergence excess is the most common or second most common binocular vision impairment (28, 33–35). It is the least common ailment, according to one survey (35). The various reports' adoption of different diagnostic criteria is believed to be the primary cause of this disagreement. The prospective design and the use of multiple sign categorization for diagnosis are the strengths of the current research.

Cycloplegic refraction cannot be done in a school because the instillation of eye drops at school set-up is not permitted by existing healthcare protocols in many countries (37–38). Participants wore complete habitual corrections throughout the experiment. Further research or reports on binocular vision activity in various refractive error groups in Indian adolescents should be conducted due to variances in the proportion of binocular vision impairments in each refractive error grouping. Understanding the aetiology of the relationships discovered in the present study may be aided by further studies in this field. The diagnosis is standardized by using a multiple-sign categorization system; otherwise, the specifics would vary widely across investigations (6, 14). There should be an effort made to combine evidence-based diagnostic standards with uniform cut-off values, testing techniques, including instruments, even testing training, and fixation targets.

As accommodative and vergence disorders are among the most prevalent visual disorders associated with myopia, this research underlines the necessity for early diagnosis of binocular vision or accommodative disorder among myopia and highlights the incidence of non-strabismic binocular vision abnormalities. Our data reinforce the practitioner who has been active in this area of vision care to assess binocular vision disorder as at least one type of binocular vision disorder found to be associated with myopia refractive error, so that proper refractive correction, vision therapy can be advised and follow-up should be done for further care.
